# Erratum: Type 1 regulatory T cell-mediated tolerance in health and disease

**DOI:** 10.3389/fimmu.2022.1125497

**Published:** 2023-01-24

**Authors:** Frontiers Production Office

**Affiliations:** Frontiers Media SA, Lausanne, Switzerland

**Keywords:** type 1 regulatory T (Tr1) cells, immunological tolerance, autoimmunity, inflammatory bowel disease, infectious disease

Due to a production error, there was a mistake in **Table 1** as published. Some of the citations in **Table 1** did not link to the correct references in the reference list. The corrected **Table 1** appears below. The publisher apologizes for this mistake.

**Table 1:** Overview of the role of Tr1 and IL-10-producing CD4^+^ T cells in AIDs and IBD.

Disease	Cell identity	Model	Major finding	Reference
***T1D***	FOXP3^-^ IL-10-producing CD4^+^ T cells	NOD BDC2.5 TCR-transgenic and NOD/SCID mice	Intestinal FOXP3^-^ IL-10-producing CD4^+^ T cells can protect from T1D	76
	IL-10-producing CD4^+^ T cells	NOD and NOD/SCID mice	Combination of rapamycin and IL-10 reduces incidence of diabetes	77
	GAD65-specific IL-10-producing CD4^+^ T cells	GAD65-immunized NOD and NOD/SCID mice	Protection from T1D in adoptive transfer experiments	78
	Antigen-specific IL-10-producing CD4^+^ T cells	Immunized NOD and NOD/SCID mice	Antigen-containing PLG nanoparticles protect from T1D	79
	CD49b^+^LAG-3^+^ Tr1 cells	Immunized NOD and NOD/SCID mice	IGRP- and 2.5mi/IA^g7^ nanoparticles expand pre-existing Tr1 cells and protect from T1D	80
	Islet-specific IL-10-producing CD4^+^ T cells	Patient PBMCs	Higher frequency in healthy donors	81
	Islet-specific IL-10-producing CD4^+^ T cells	Patient PBMCs	Higher frequency in first degree relatives	82
	Insulin-specific IL-10-producing CD4^+^ T cells	Patient PBMCs	Higher in patients that have better future glucose control	83
	IGFR-specific IL-10-producing CD4^+^ T cells	Patient PBMCs	Higher frequency in juvenile T1D compared to adult T1D	84
	IL-10-producing CD4^+^ T cells	Patient PBMCs	Increase in IL-10-producing CD4^+^ T cells upon anti-CD3 treatment	85
	Proinsulin-specific IL-10-producing CD4^+^ T cells	Proinsulin-immunized patient PBMCs	Increase in IL-10 production and decrease in insulin dependency	86
	IGRP-specific CD49b^+^LAG-3^+^ Tr1 cells	IGRP-immunized NSG mice reconstituted with DR4 patient PBMCs	Induction of IGRP-specific CD49b^+^LAG-3^+^ Tr1 cells	80
***MS***	OVA-specific IL-10-producing CD4^+^ T cells	OVA-immunized MSCH-induced EAE mice	OVA-specific protection from EAE development	87
	IL-10-producing CD4^+^ T cells	MOG-induced EAE mice	IL27-pulsed DCs upregulate IL-10 production by CD4^+^ T cells for protection from EAE development	88
	CD49b^+^LAG-3^+^ Tr1 cells	MOG-induced EAE mice	Protection from EAE development independently of IL-10	89
	IL-10-producing CD4^+^ T cells	MOG-induced EAE mice	Melatonin induces IL-10-producing CD4^+^ T cells to protect from EAE development	65
	CD49b^+^LAG-3^+^ Tr1 cells	PLP-induced EAE mice	PLP- and MOG-containing pMHCII nanoparticles protect from EAE development	80
	IL-10-producing CD4^+^ T cells	PLP- and MOG-induced EAE	MOG- and AhR-containing NLPs protects from EAE development	90
	CD46-induced IL-10-producing CD4^+^ T cells	Patient PBMCs	Lower frequency in MS patients	91
	CD46-induced IL-10-producing CD4^+^ T cells	Patient PBMCs	Altered glycosylation of CD46 in MS patients	57
***SLE***	CD46-induced IL-10-producing CD4^+^ T cells	Patient PBMCs	Lower frequency in SLE patients	24
	IL-10-producing IL7R^-^ CD4^+^ T cells	Patient PBMCs	Decreased ability in limiting autoantibody production by B cells	16
	IL-10-producing CXCR5^-^CXCR3^+^PD-1^high^ CD4^+^ T cells	Patient PBMCs	CXCR5^-^CXCR3^+^PD-1^high^ CD4^+^ T cell-derived IL-10 can stimulate autoantibody production	92
***Arthritis***	Collagen type II-specific IL-10-producing CD4^+^ T cells	Collagen type II-immunized DBA/1 mice	Protection from arthritis development	93
***Psoriasis***	CD49b^+^LAG-3^+^ Tr1 cells	Patient PBMCs and skin biopsies	Presence of Tr1 cells inversely correlated with disease severity	94
***Celiac disease***	Gliadin-specific IL-10-producing CD4^+^ T cells	Patient intestinal T cells	Gliadin-specific suppression of effector T cells	44
	Gliadin-specific IL-10-producing CD4^+^ T cells	Patient PBMCs and intestinal T cells	Multi-epitope gliadin extract induces tolerance in celiac disease patients	95
***IBD***	OVA-specific IL-10-producing CD4^+^ T cells	CD4^+^CD45RB^high^ adoptive transfer mice	Prevention of colitis in recipient mice	4
	IL10-deficient CD4^+^CD45RB^low^ T cells	CD4^+^CD45RB^high^ adoptive transfer mice	Protection from colitis is dependent on IL-10	96
	IL-10-producing CD4^+^ T cells	CD4^+^CD45RB^high^ adoptive transfer mice	Prevention of Th17-mediated colitis	97
	IL-10-producing CD4^+^ T cells	DSS-induced colitis mouse model	GSK-J4-mediated induction of tolerogenic DCs to promote IL-10 production by CD4^+^ T cells	98
	IL-10-producing CD44^+^CD4^+^ T cells	TNBS-induced colitis mouse model	MSCs induce expansion of IL-10-producing CD44^+^CD4^+^ T cells	99
	Intestinal IL7R^-^CCR5^+^PD-1^+^ Tr1 cells	Patient intestinal T cells	Decreased IL-10 production	30
	IL-10-producing OVA-Treg	Patient PBMCs	Amelioration of disease in treated patients	100
	CD49b^+^LAG-3^+^ Tr1 cells	Patient PBMCs and colon biopsies	Decrease of myeloid-derived inflammatory cytokines and production of IL-22 and mucin to promote barrier integrity	69

The original version of this article has been updated.
